# 615. Utilizing Susceptible-Infected-Recovered (SIR) Modeling in Colorado Schools and Childcare Facilities to Identify Schools Most Susceptible to Measles Outbreaks

**DOI:** 10.1093/ofid/ofaf695.188

**Published:** 2026-01-11

**Authors:** Paul G Mitchell, Rachel L Schade, Jessica R Cataldi, Carl Amon, James Todd, Edwin J Asturias

**Affiliations:** Children's Hospital Colorado, Aurora, CO; Children's Hospital Colorado, Aurora, CO; University of Colorado School of Medicine, Aurora, Colorado; Children's Hospital Colorado, Aurora, CO; University of Colorado/Children's Hospital Colorado, Aurora, CO; CU School of Medicine, Aurora, Colorado

## Abstract

**Background:**

Pockets of unvaccinated communities leave children vulnerable, even in areas with overall high vaccination rates. Schools are one of the most susceptible environments for measles outbreaks. This study used 2023-2024 Colorado School & Child Care Immunization Data to predict potential measles outbreaks in schools, providing actionable information to guide prevention and mitigation strategies.

**Figure 1:**
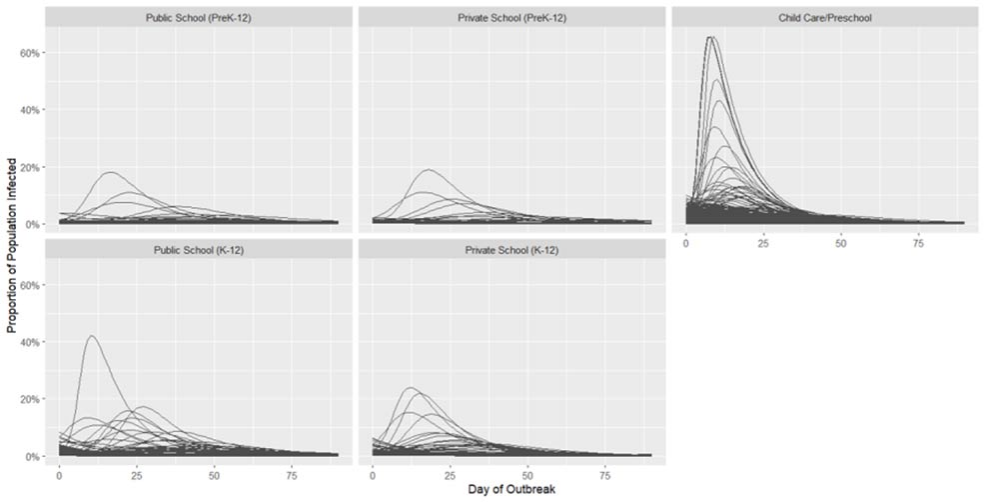
SIR Epidemic Curves of Proportion of Facility Population Infected by Day of Outbreak, Stratified by Type of Facility

**Table 1:**
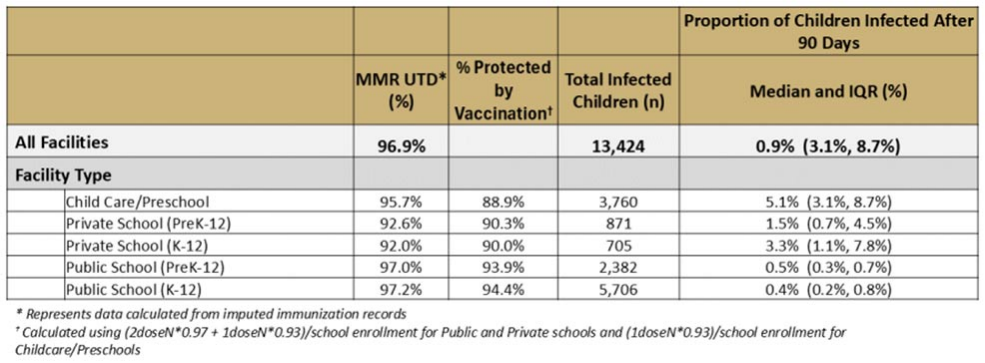
MMR Up-To-Date Rates and Proportion of Children Infected at Each School, Stratified by Type of School

**Methods:**

School-level immunization data was drawn from Colorado Department of Public Health & Environment annual reports. We developed a deterministic, compartmental Susceptible Infected Recovered model to simulate spread within schools following the introduction of 1 measles case. School-aged children with two MMR doses were estimated to have 97% protection, whereas children in schools or childcare centers with one dose were estimated to have 93% protection. We assumed a fixed contact rate of 95% and recovery rate of 10% (i.e. R₀ = 9.5) for each infection during the outbreak with a 90 day duration.

**Figure 2:**
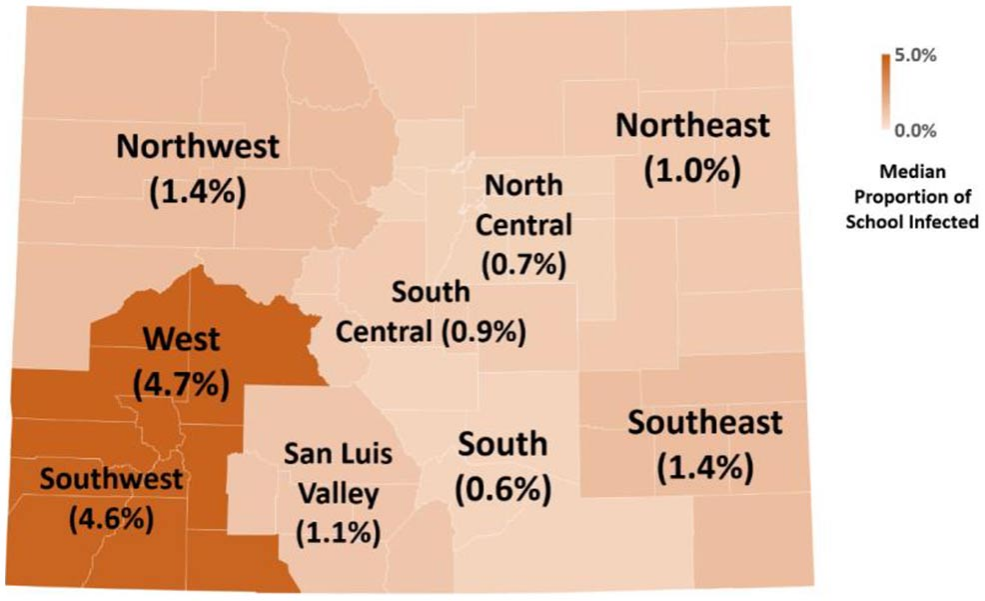
Median Proportion of Infected Students after 90-day Simulated Outbreak Among CO Schools in 9 Department of Homeland Security All-Hazard Regions

**Figure 3:**
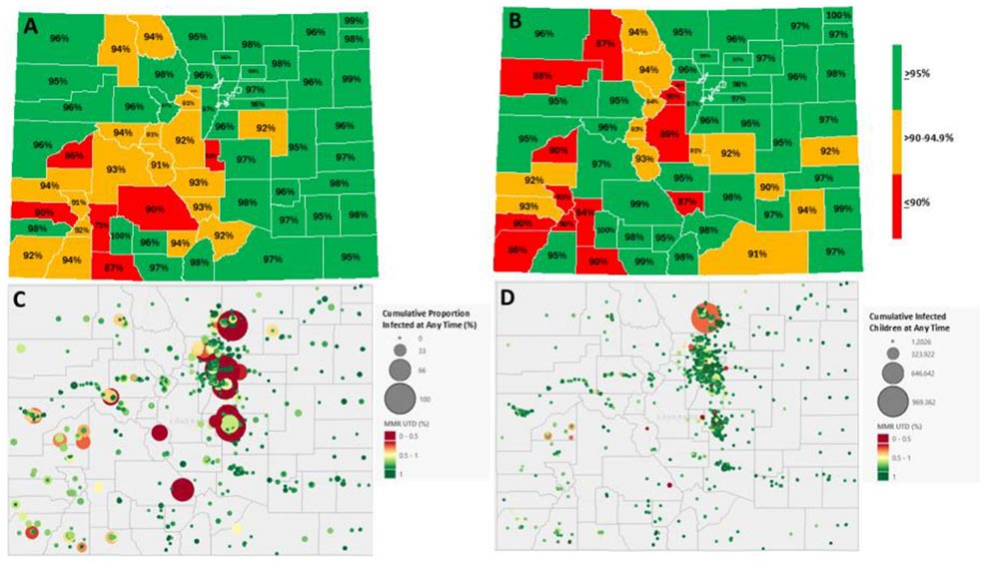
Maps of MMR Vaccination Rates and Modeled Measles Outbreaks Across Colorado A. 2023-2024 Colorado K-12 Schools 2 Dose MMR Up-To-Date % by County B. 2023-2024 Colorado Childcare/Preschool 1 Dose MMR Up-To-Date % by County C. Geospatial Colorado Statewide Plot of Simulated School Outbreaks by MMR Up-to- Date Rate and Proportion of School Infected by End of 90 Day Outbreak D. Geospatial Colorado Statewide Plot of Simulated School Outbreaks by MMR Up-to- Date Rate and Total Number of Infected Children by End of 90 Day Outbreak

**Results:**

Simulated outbreak size in 2,524 Colorado facilities ranged from 0.04-99.99% of the specific school population (Figure 1). Proportion infected over the outbreak differed between school types with highest median outbreak size in childcare centers (Table 1). 71% of infections in childcare centers occurred in the first 14 days of the outbreak with only 33-35% in K-12 schools during that time. Median outbreak size also differed across state regions from a median of 0.6% infected in the South to 4.7% in the West region (Figure 2). When plotted geospatially, outbreaks infecting large portions of the facility occurred across the state, even in well vaccinated counties (Figure 3).

**Conclusion:**

Childcare centers are more susceptible to larger and more rapid outbreaks compared to K–12 schools, underscoring the need for prompt mitigation strategies. Private K–12 schools are more at risk relative to public schools, likely due to higher vaccine exemption rates. Notably, large potential outbreaks were observed even in the densely populated Denver Metro area, despite high overall immunization coverage. These findings highlight the limitations of using aggregated state or county-level vaccination data to assess outbreak risk and indicate that children in and around affected schools remain vulnerable.

**Disclosures:**

All Authors: No reported disclosures

